# Obstructive sleep apnea risk is associated with poor physical performance: a cross-sectional analysis of the U.S. health and retirement study

**DOI:** 10.1007/s44470-026-00070-2

**Published:** 2026-04-07

**Authors:** Rajani Khanal, Kimberley S. van Schooten, Ronaldo Piovezan, Robert Adams, Kelly Sansom, Andrew Vakulin

**Affiliations:** 1 Flinders Health and Medical Research Institute, Sleep Health, Flinders University, Adelaide, South Australia 5042 Australia; 2 Falls, Balance and Injury Research Centre, Neuroscience Research Australia, Sydney, NSW Australia; 3 School of Population Health, University of New South Wales, Sydney, NSW Australia; 4 Adelaide Geriatrics Training and Research With Aged Care (GTRAC) Centre, University of Adelaide, Adelaide, South Australia Australia; 5 Department of Geriatrics and Rehabilitation Medicine, Royal Adelaide Hospital, Central Adelaide Local Health Network, SA Health, Adelaide, South Australia Australia; 6 Centre for Healthy Ageing, Health Futures Institute, Murdoch University, Murdoch, WA Australia

**Keywords:** Balance, Gait, Handgrip strength, Falls, Sleep apnea

## Abstract

**Study objectives:**

Obstructive sleep apnoea (OSA) may be linked to poor physical performance and fall risk, yet this association remains underexplored. This study examined associations between OSA risk, balance, gait speed and handgrip strength (HGS) in community living adults across age-groups and sexes.

**Methods:**

Cross-sectional data from the 2016 Health and Retirement Study were analysed. Probable OSA was estimated with an adapted STOP-Bang questionnaire. Poor balance was defined as the inability to hold a semi-tandem stance for 10 s; slow gait speed as walking < 0.8 m/s over 2.5 m; and weak HGS as HGS-to-body mass index ratio < 1.00 m^2^ for males and < 0.56m^2^ for females.

**Results:**

6,918 participants (mean age 66 ± 11 years; 57% female) were included. Probable OSA was associated with higher odds of: (i) poor balance in the overall sample (OR:1.23, 95% bootstrapped confidence interval (BCI):1.07–1.39, *p* = 0.002), 50–64 years (OR: 1.41, BCI: 1.15- 1.72, *p* < 0.001) and females (OR: 1.30, BCI: 1.10–1.56, *p* = 0.004); (ii) slow gait speed in the overall sample (OR:1.29, BCI:1.07–1.57, *p* = 0.007), 80 + years (OR:1.61, BCI:1.07–2.42, p = 0.028) and females (OR:1.39, BCI:1.03–1.91, *p* = 0.024); and (iii) weak HGS in the overall sample (OR:2.22, BCI:1.90–2.63, *p* = 0.001), 50–64 years (OR:3.40, BCI: 2.58–4.61, *p* < 0.001), 65–79 years (OR: 1.93, BCI:1.52- 2.47, *p* < 0.001), males (OR = 1.87, BCI:1.49–2.35, p < 0.001) and females (OR = 2.67, BCI 2.15–3.33, *p* < 0.001).

**Conclusions:**

Poor balance, slow gait speed and weak HGS are common among older adults at high risk of OSA. Further research should evaluate causality and assess co-screening to potentially enable early detection of fall risk in older adults.

**Brief summary:**

**Study Rationale:** OSA is a common but often undiagnosed condition that may contribute to accelerated age-related physical decline and increased fall risk. Despite known links between diagnosed OSA and motor deficits, little is known about how undiagnosed OSA relates to fall-related physical performance measures in large, community-based populations.

**Study Impact:** This study suggests that individuals at high risk of OSA are more likely to have poor balance, slow gait speed, and weak handgrip strength, which are key predictors of fall risk. The observation of these associations in adults as young as 50 years of age warrants future research to evaluate causality and determine if co-screening of OSA and fall risk can help identify those most vulnerable.

**Supplementary Information:**

The online version contains supplementary material available at 10.1007/s44470-026-00070-2.

## Introduction

Falls are a major global health issue, with an estimated age-standardised incidence of 2,238 per 100,000 in 2017 [1]. Among older adults, falls rank as the second highest cause of injury-related deaths [2]. A primary contributor to falls in this population is the age-related decline in physical performance, especially impairments in balance, gait and muscle strength [3–5]. Poor balance increases postural sway and instability during dynamic tasks, such as walking, turning, or navigating uneven surfaces [6]. Impaired gait patterns, characterised by shorter, more variable steps and slower walking speed increase the likelihood of tripping or falling [7, 8]. Relative handgrip strength (HGS), calculated as the ratio of HGS to body mass index (BMI), is a useful indicator of muscle weakness [9] and is associated with increased fall risk [10]. Together, measures such as standing balance, gait speed and relative HGS, serve as objective indicators of fall risk and help identify individuals most vulnerable to falls.

Emerging evidence suggests that obstructive sleep apnea (OSA) may also contribute to fall risk in older adults [8]. Prevalence of OSA increases markedly with age affecting nearly half of adults aged 60 and older [11]. OSA disrupts normal sleep patterns and leads to intermittent nocturnal hypoxemia, which can negatively impact sensorimotor and cognitive systems that are essential to maintain stability and coordination, and thereby compromise mobility and increase fall risk [8]. Experimental studies that examine associations of OSA and postural balance have found worse balance among individuals with OSA [12–15], with evidence that postural stability in those with severe OSA is worse than that of those with non-severe OSA [16]. Similarly, experimental studies which examine associations of OSA and gait impairments showed that individuals with OSA exhibit increased step width and decreased stride length [17], greater stride-to-stride variability [18], and slow gait speed [19], all indicators of poor balance during walking. Fewer studies have examined the associations of OSA and muscle strength and, those that have, had inconsistent results [20, 21]. Notably, a cohort study on 2,862 community-living males aged 65 years and older revealed that individuals with OSA had weak HGS and slower gait speed [22].

These findings are particularly relevant because, although OSA prevalence increases with age and often co-occurs with poor physical performance, up to 80% of OSA cases may remain undiagnosed [23]. Given the importance of preventing physical decline before it leads to falls, early identification of potentially modifiable risk factors such as OSA may offer critical opportunities for intervention. This highlights the need to consider not only diagnosed cases but also individuals at high risk of OSA, who may already show early signs of poor physical performance. However, no study to date has examined the associations between OSA risk and multimodal measures of fall-related physical performance in a large, representative sample. Moreover, it remains unclear whether these associations differ by age or sex.

To address these gaps, this study aimed to examine the associations between probable (high-risk) OSA and standing balance, gait speed, and relative HGS, using data from a large, nationally representative sample of adults over 50 years of age from the Health and Retirement Study. We also conducted age- and sex-stratified analyses to explore potential subgroup differences. We hypothesised that individuals at high risk of OSA would have poor physical performance.

## Methods

### The health and retirement study

We utilised data from the 2016 wave of the Health and Retirement Study (HRS). Information on data collection methodologies, participant characteristics and selection criteria has been published in detail elsewhere [24–26]. Briefly, the HRS is a nationally representative, longitudinal study conducted by the University of Michigan’s Institute for Social Research, sponsored by the National Institute on Aging (U01AG009740). The study began in 1992 and includes successive birth cohorts to maintain representativeness of the community-living, non-institutionalised U.S. population aged 50 years and above, also including their partners or spouses (who may be less than 50 years old). The study collects data biennially through face-to-face and telephone interviews. In 2006, a random half of the sample was assigned to enhanced face-to-face interviews where they were asked to complete the physical performance measures, including standing balance, gait speed, and handgrip strength testing. The other half of the sample was assigned to the enhanced face-to-face in the next wave (2008), such that moving forward, physical performance measures are available every 2 years on half the sample of participants only.

The study is approved by University of Michigan Institutional Review Board (IRB0000921). All participants provided written consent for anonymous use of their public survey data.

### Study sample

**Fig. 1:**
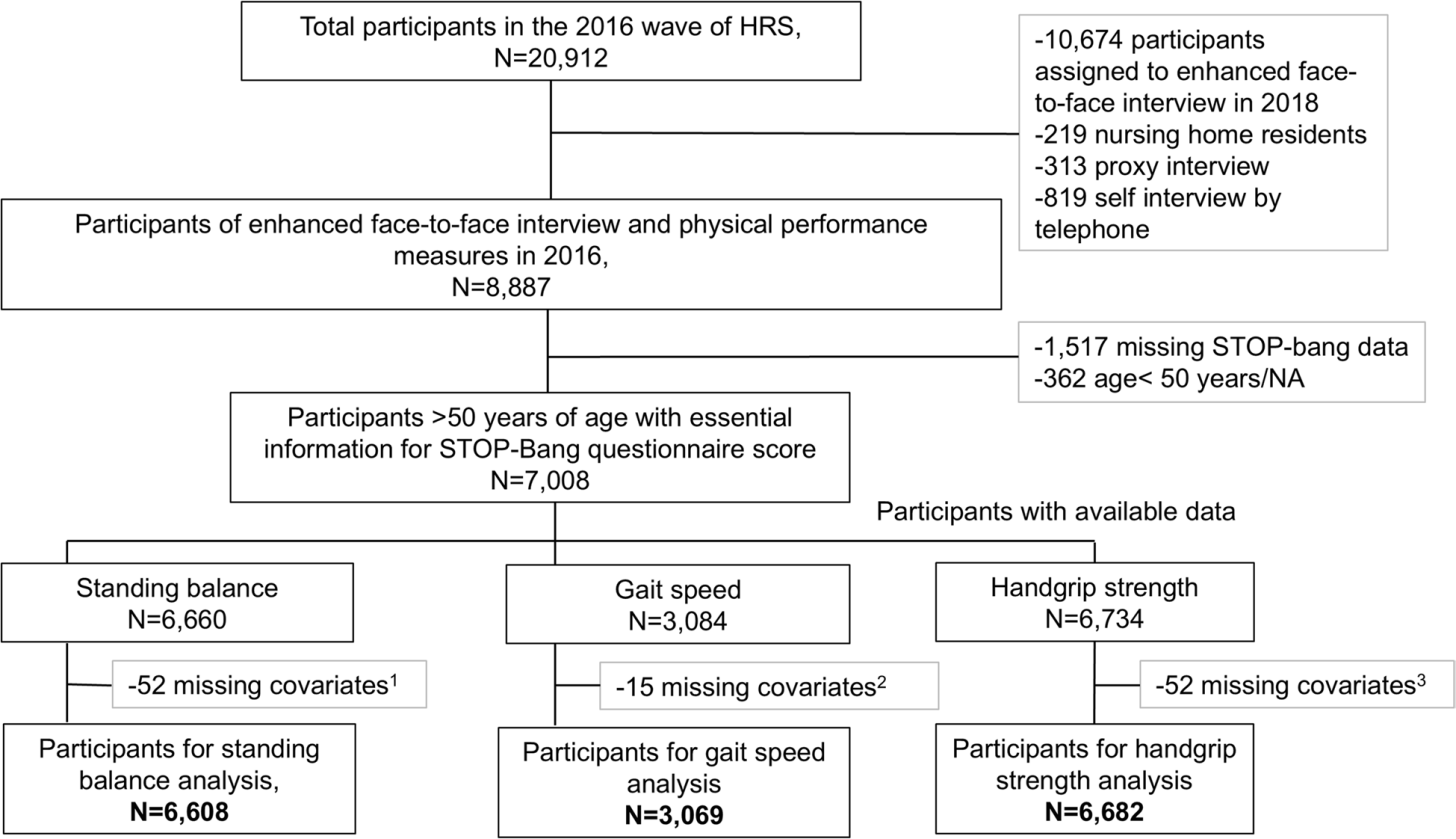
Participant selection flowchart. Missing covariates^1^: −21 missing race/ethnicity data, −2 missing alcohol consumption data, −30 missing physical activity data, Missing covariates^2^: −2 missing race/ethnicity data, −13 missing physical activity data, Missing covariates^3^: −22 missing race/ethnicity data, −2 missing alcohol data, −29 missing physical activity data

For the current analysis, we used the HRS 2016 wave, since questions regarding sleep, including those aligning with the STOP-Bang questionnaire [27], were first introduced in this year. We limited the study sample to the respondents who: (1) were selected for physical performance measures via enhanced face-to-face interviews in the 2016 wave, (2) were aged 50 years and above, (3) had non-missing answers for the STOP-Bang questionnaire, and, (4) had information on at least one of the balance, gait, or handgrip strength measurements. A total of 8,887 people participated in the enhanced face-to-face interviews and physical performance measures. Out of these, 7,008 participants had data on the STOP-bang questionnaire items and were aged above 50 years. Gait speed (m/s) was measured only among people 65 years and older [26]. In total, 6,602 participants had data on standing balance, 3,069 on gait speed and 6,675 on handgrip strength, along with necessary covariates: age, sex, race/ethnicity, height, weight, smoking status, alcohol consumption, comorbidities, treatment for sleep or snoring problems, and physical activity levels (Fig. 1).

### Exposure: obstructive sleep apnoea risk

The STOP-Bang questionnaire is a widely validated screening tool for OSA risk, comprising eight items: snoring, tiredness, observed apnea, high blood pressure, BMI, age, neck circumference, and sex [27]. In the 2016 wave, the HRS included survey questions that correspond to 7 out of 8 STOP-Bang items (missing neck-circumference). In the current analysis, these survey questions were mapped to their corresponding STOP-Bang items, to define the exposure variable OSA risk (Table 1), using the methodology previously applied for HRS dataset [28–30]. In line with previous literature probable (high-risk) OSA was defined using two criteria (1) a score of ≥ 3, where at least two positive responses are from the “STOP” items and one response is from male sex and/or BMI > 35 kg/m^2^; or (2) a total score of ≥ 4 based on any combination of positive items [28, 31]. Those not meeting these criteria were categorised into low-risk group. Criterion (1) has a high specificity (97% for moderate and severe OSA) ensuring that most participants classified as high-risk truly have OSA [27]. Criterion (2) has higher sensitivity (68% for moderate OSA and 79% for severe OSA), capturing additional high-risk OSA cases that do not meet the more stringent rule [27, 31]. This approach of identifying individuals at high risk of OSA using 7 items has been used in previous population-based studies and has demonstrated effectiveness across diverse cohorts, including older adults [28–30].

**Table 1:** Mapping HRS survey questions to the corresponding STOP-Bang items

STOP-Bang items	Corresponding information from HRS	Thresholds for interpreting HRS questions as positive results
Do you snore loudly?	“In the past 12 months, how often did you snore while you were sleeping?”	Responses equivalent to “ ≥ 3 nights/week” which include Occasionally (3–4 nights/week), and frequently (5 or more nights/week)
Do you often feel tired, fatigued, or sleepy during the daytime?	“Have you had any of the following persistent or troublesome problems? Severe fatigue or exhaustion”	Yes
Has anyone observed you stop breathing during sleep?	“In the past 12 months, how often did you snort, gasp, or stop breathing while you were sleeping?”	Responses equivalent to “ ≥ 1 nights/week” which include: “Rarely (1–2 nights/week)”, “occasionally (3–4 nights per week)”, or “frequently (5 or more nights per week)”
Do you have (or are you being treated for) high blood pressure?	Has a doctor ever told you that you have high blood pressure or hypertension?” or “Prev wave has high blood pressure?”	Yes
BMI	Calculated based on the “About how much do you weigh (lbs)?” and “How tall are you (feet, inches)?”	> 35 kg/m^2^
Age	Calculated based on the “year born”	> 50 years
Neck circumference	NA	NA
Sex	Sex	Male

### Outcome measures

#### Standing balance

Standing balance was assessed using a structured three-step protocol in the HRS [26]. Participants were asked to perform a semi-tandem stance, where the side of the heel of one foot touches the side of the toe of the opposite foot, and to maintain this position for 10 s. For this study, poor balance was defined as failure to complete the semi-stand-stance and optimal balance as able to complete the semi-stand-stance [32, 33].

#### Gait speed

Participants were instructed to walk at their usual pace across a 2.5-m course and back [26]. The average duration of the two trials was used to calculate average gait speed, which was derived by dividing the distance walked (meters) by the time taken (seconds). Slow gait speed was defined as < 0.8 m/s, and optimal gait speed was defined as gait speed ≥ 0.8 m/s, based on established clinical thresholds [34].

#### Relative HGS

HGS was measured using a Smedley spring-type hand dynamometer (Scandidact; Odder, Denmark). Participants with recent surgery, pain, or injury in both hands were excluded, but if symptoms affected only one hand, the other was used with a 30-s rest between trials. During the test, participants were instructed to squeeze the device as hard as possible for a few seconds and then release. Participants first completed a practice trial with their dominant hand while standing with their arm at a 90-degree angle; if they were unable to stand, they could remain seated. If a participant had difficulty holding the device, support was provided by allowing their arm to rest on a table. Two alternating measurements were taken for each hand (four in total), with the interviewer recording each result. The maximum value from the four measurements was used as the participant’s HGS before computing relative HGS as the ratio of maximum HGS to BMI [35]. Weak HGS was defined as relative HGS < 1.00m^2^ for males and < 0.56m^2^ for females [36].

### Covariates

Sociodemographic information on age, sex, ethnicity, smoking status, alcohol consumption, comorbidities, treatment for sleep or snoring problems, and physical activity was collected via structured interviews. Height and weight were measured during the enhanced face-to-face interview.

Height was measured without shoes in inches. Weight was measured in pounds using a Healthometer 830KL digital scale. Body mass index (BMI) was calculated by first converting height from inches to meters and weight from pounds to kilograms, then applying the standard formula: weight (kg) divided by height (m^2^).

Ethnicity was categorised into four groups: Hispanic, and non-Hispanic Black, White and Other race. Smoking status was determined by the question “Do you smoke cigarettes now?”, with “yes” classified as a smoker and “no” as a non-smoker. Alcohol use was assessed by asking, “Do you ever drink any alcoholic beverages such as beer, wine, or liquor?”, with responses categorised as drinker (“yes”) or non-drinker (“no”).

Comorbidities included self-reported doctor diagnoses of heart disease, lung disease, arthritis, depression, and diabetes. A comorbidity index was created by summing the number of these conditions. A score of ≥ 2 was classified as high comorbidity burden, while a score of ≤ 1 was categorised as low burden [37].

Participants were asked, “In the past year have you had any treatments for your [sleep/snoring] problem?”. Treatment included: “oxygen, positive air pressure (PAP) device such as a continuous PAP or bilevel PAP, surgery of the nose or throat, a device to help position your jaw, nerve stimulation of the tongue, adhesive strips with or without medication, medication, and other treatments (specify)”. Those answering “yes” were considered treated for their sleep/snoring problem while those answering “no” or “don’t know” were considered untreated.

Physical activity was assessed using self-reported questions on the frequency of vigorous and mild activities: “How often do you take part in sports or activities that are vigorous, such as running or jogging, swimming, cycling, aerobics or gym workout, tennis, or digging with a spade or shovel?” and “How often do you take part in sports or activities that are mildly energetic, such as vacuuming, laundry, home repairs?” Response options for both questions included “never,” “less than once per month,” “1–3 times per month,” “1–2 times per week,” and “ ≥ 3 times per week.” We calculated a continuous leisure time physical activity (LTPA) score, by assigning point values on the reported frequency of activity adapting a previously used method by Wen and colleagues [38]. For vigorous activity, scores were 0 for “never or less than once per month”; 2 for “1–3 times per month,” 6 for “1–2 times per week,” and 12 for “three or more times per week”. For light activity, half the points assigned for vigorous activity were used (i.e., 0, 1, 3, and 6, respectively). The LTPA score (0–18) was calculated as the sum of points from both activity types and used as a continuous variable [38].

### Statistical analysis

Statistical analysis was performed in RStudio (RStudio Team 2018, Boston MA, USA) with R version 4.3.2 (R Core Teams 2021, Vienna, Austria). Normality of continuous variables was examined using histograms. Participant characteristics were summarised using means and standard deviations (SD) for normally distributed continuous variables, median with interquartile range (IQR) for non-normally distributed continuous variables and counts with percentages for categorical variables. Independent samples t-tests, Wilcoxon rank sum test and Pearson’s χ2 test of homogeneity were used to compare participants’ characteristics according to OSA risk. P-values of less than or equal to 0.05 were considered statistically significant.

Binomial logistic regression analyses examined associations of OSA risk as an independent variable with standing balance, gait speed and relative HGS as dependent variables. Models for poor balance and slow gait speed were adjusted for age, sex, BMI, race, alcohol consumption, comorbidity burden, treatment for sleep/snoring problem and LTPA. The model for relative HGS was not adjusted for BMI since the outcome already included BMI. All models were further examined using age and sex-stratified analyses. Bootstrapped confidence intervals (BCI;95%) were used to improve the precision and reliability of the estimated association using the boot package [39]. A sensitivity analysis was performed by excluding participants who reported prior treatment for sleep or snoring problems (Supplementary Material S1).

## Results

### Participant characteristics

Table 2 shows participant characteristics by OSA risk. A total of 6,918 participants were included in the analysis, with 4,176 (60%) classified as low-risk and 2,742 (40%) as probable (high-risk) OSA based on the STOP-Bang score.

**Table 2:** Participant characteristics

Characteristics	Overall (*N* = 6918)^1^	Low-risk OSA (*N* = 4176)^1^	Probable (high-risk) OSA (*N* = 2742)^1^	*p*-value^2^
Male (%)	2,972(43%)	1,342 (32%)	1,630 (59%)	** < 0.001**
Age (years)	66 (11)	66 (11)	64 (10)	** < 0.001**
BMI (kg/m^2^)	30 (7)	28 (6)	33 (7)	** < 0.001**
Race/ethnicity^3^ (%)				** < 0.001**
White	4,558 (66%)	2,836 (68%)	1,722 (63%)	
Black	1,486 (21%)	821 (20%)	665 (24%)	
Hispanic	298 (4.3%)	180 (4.3%)	118 (4.3%)	
Other^4^	576 (8.3%)	339 (8.1%)	237 (8.6%)	
Leisure time physical activity (LTPA 0–18)	6.0 (3.0–12.0)	6.0 (3.0–15.0)	6.0 (3.0–12.0)	** < 0.001**
Drinker (%)	4,221 (61%)	2,527 (61%)	1,694 (62%)	0.302
High comorbidity burden (%)	3,367 (49%)	1,790 (43%)	1,577 (58%)	** < 0.001**
Treated for snoring (%)	596 (8.6%)	229 (5.5%)	367 (13%)	** < 0.001**
Poor balance (%)	2,112 (32%)	1,242 (31%)	870 (34%)	**0.018**
Slow gait speed (%)	1,927 (63%)	1,210 (61%)	717 (66%)	**0.018**
Weak relative HGS (%)	1,009 (15%)	445 (11%)	564 (21%)	** < 0.001**

^1^n (%); Mean (SD); Median (IQR); Bold values indicate statistical significance (p<0.05)

^2^Pearson’s Chi-squared test; Welch Two Sample t-test; Wilcoxon rank sum test

^3^White race is the reference; ^4^Other include American Indian, Alaskan Native, Asian, Pacific Islander and unascertained

There were more males in the probable OSA group compared to females and they had a higher mean BMI compared to those in the low-risk group. The mean age was slightly lower in the probable OSA group than in the low-risk group. A higher proportion of people reporting African American ethnicity were in the probable OSA group. Participants in the probable OSA group also had a significantly higher comorbidity burden compared to the low-risk group. Treated sleep or snoring problems were more common in the probable OSA group though the majority in both groups were untreated. Leisure time physical activity levels were similar among the probable and low-risk group. No significant difference was found in alcohol consumption between groups. In terms of physical performance, poor balance was more common in the probable OSA group, as were slow gait speed and weak relative HGS.

### Associations of OSA risk with balance, gait and relative HGS

**Fig. 2:**
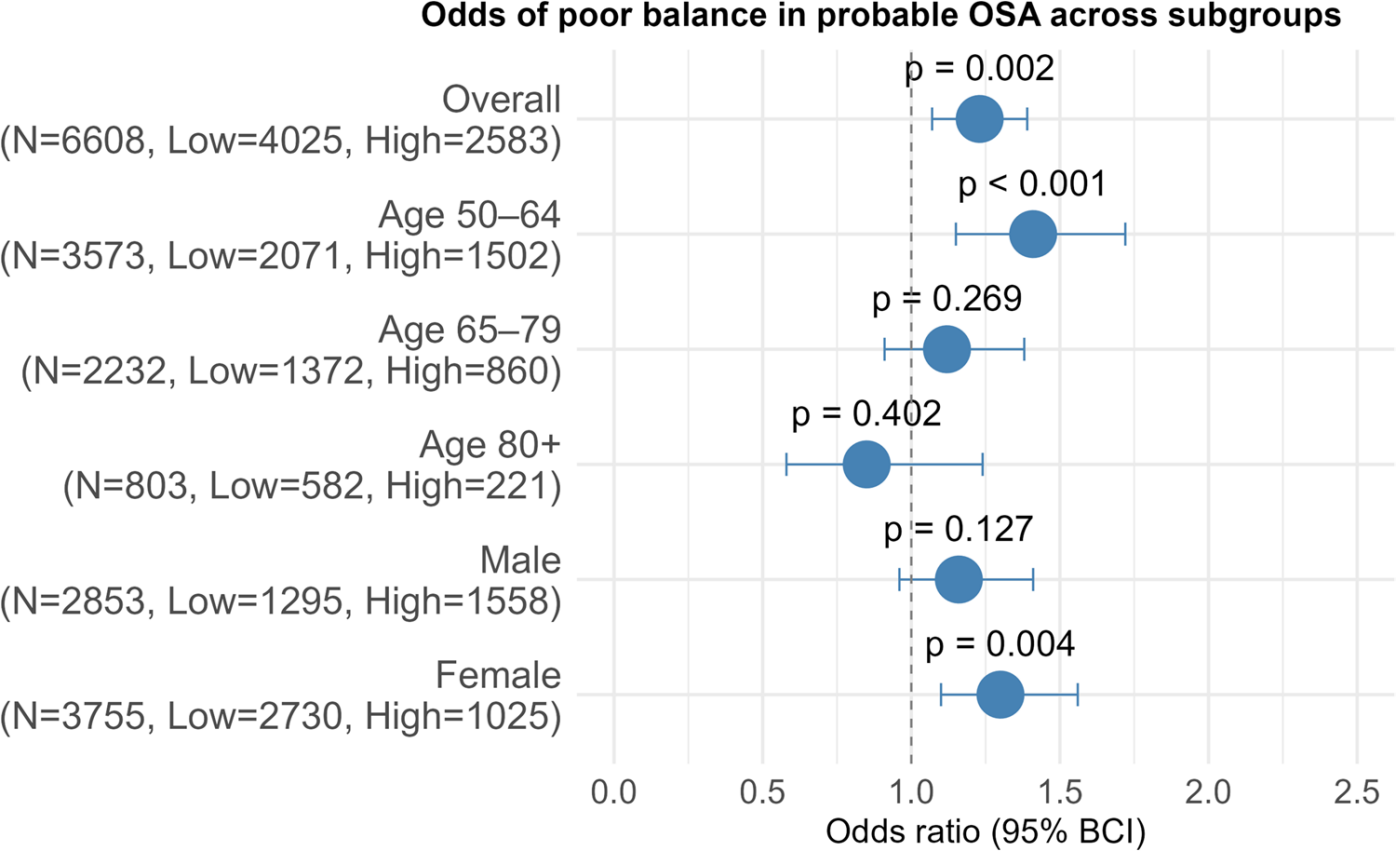
Odds of poor balance in probable OSA across subgroups. Overall model is adjusted for age, sex, BMI, race, alcohol consumption, comorbidity burden, treatment for sleep/snoring problem and LTPA. N= Total number of participants in the subgroup, Low= Number of participants with low-risk OSA in the subgroup, High= Number of participants with probable (high-risk) OSA in the subgroup

**Fig. 3:**
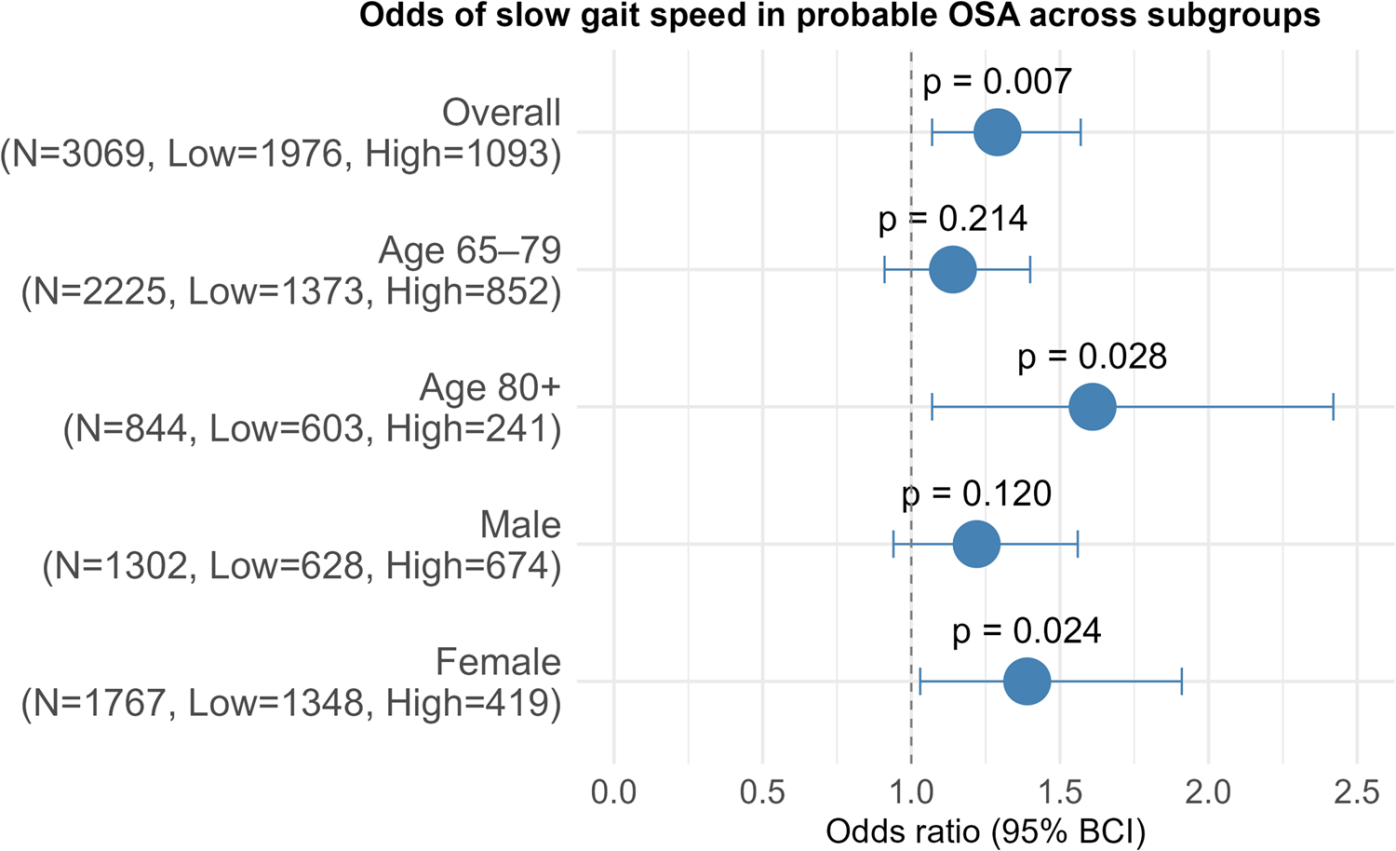
Odds of slow gait speed in probable OSA across subgroups.Overall model is adjusted for age, sex, BMI, race, alcohol consumption, comorbidity burden, treatment for sleep/snoring problem and LTPA. N= Total number of participants in the subgroup, Low= Number of participants with low-risk OSA in the subgroup, High= Number of participants with probable (high-risk) OSA in the subgroup

**Fig. 4:**
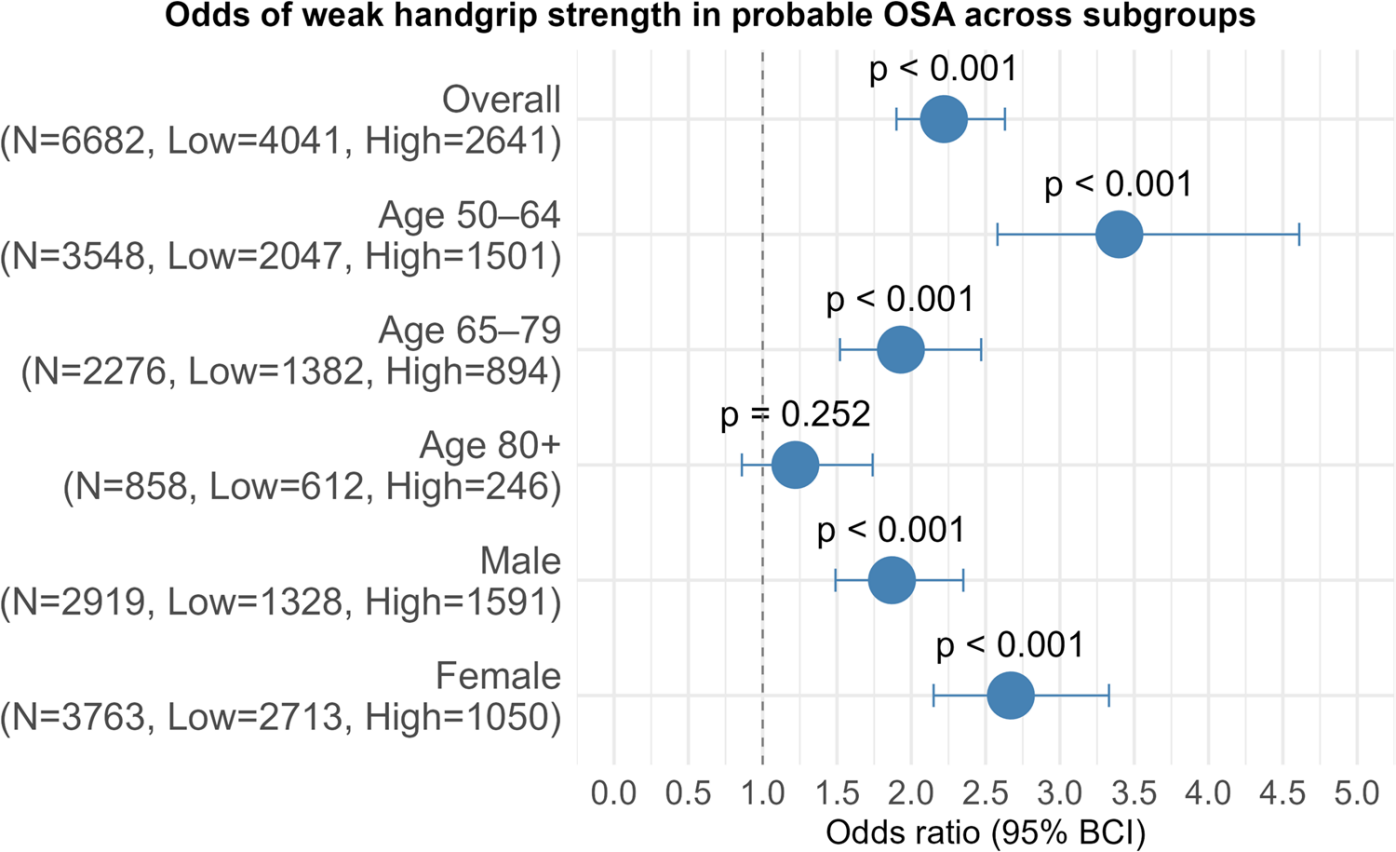
Odds of weak relative HGS in probable OSA across subgroups. Overall model is adjusted for age, sex, race, alcohol consumption, comorbidity burden, treatment for sleep/snoring problem and LTPA. N= Total number of participants in the subgroup, Low= Number of participants with low-risk OSA in the subgroup, High= Number of participants with probable (high-risk) OSA in the subgroup

Probable OSA was significantly associated with higher odds of poor balance (OR: 1.23, 95% BCI: 1.07–1.39, *p* = 0.002), slow gait speed (OR: 1.29, 95% BCI: 1.07–1.57, *p* = 0.007), and weak HGS (OR: 2.22, 95% BCI: 1.90–2.63, *p* < 0.001) after adjusting for confounders (Figs. 2, 3 and 4). Sensitivity analyses excluding participants treated for sleep/snoring problems yielded consistent results with the main analyses (Supplementary Material S1).

### OSA and balance stratified by age and sex

Figure 2 shows the odds of poor balance in the probable OSA group across different age groups and sexes. The association between probable OSA and poor balance was strongest in the 50–64 years age group, with 41% increased odds of poor balance (OR: 1.41, BCI: 1.15- 1.72, *p* < 0.001). The association was weaker in the 65–79 years age group (OR:1.12, 95% BCI: 0.91–1.38, *p* = 0.269) and in the 80 + years age group (OR: 0.85, BCI: 0.58–1.24, p = 0.402), where the wide BCI indicates uncertainty of the estimate. For males, the association between probable OSA and poor balance was not statistically significant (OR: 1.16, BCI: 0.96–1.41, *p* = 0.127), but more compatible with a moderate harmful effect than a protective effect. For females, probable OSA was significantly associated with a 30% increased odds of poor balance (OR: 1.30, BCI: 1.10–1.56, *p* = 0.004).

### OSA and gait stratified by age and sex

Figure 3 shows the odds of slow gait speed in the probable OSA group across different age groups and sexes. There was no gait speed data available for people aged 50–64 years. In the age group 65–79 years, the data are more compatible with a small to moderate harmful effect of probable OSA on gait speed rather than a protective effect (OR: 1.14, BCI: 0.91–1.40, *p* = 0.214). In the 80 + years age group, probable OSA was significantly associated with a 61% increase in odds of slow gait speed (OR:1.61, BCI:1.07–2.42, *p* = 0.028). Similarly, for males (OR:1.22, BCI:0.94–1.56, *p* = 0.120), the confidence interval indicate compatibility with a small to moderate harmful effect of probable OSA on gait speed rather than a protective effect. In females, probable OSA was significantly associated with 39% higher odds of slow gait speed (OR:1.39, BCI:1.03–1.91, *p* = 0.024).

### OSA and relative HGS stratified by age and sex

Figure 4 shows odds of weak HGS in the probable OSA group across different age groups and sexes. The association between probable OSA and weak HGS was strongest in the 50–64 years age group, with a more than 3 folds increase in odds of weak HGS (OR:3.40, BCI: 2.58–4.61, *p* < 0.001). In the age group 65–79 years, probable OSA was associated with a 93% increased odds of weak HGS (OR: 1.93, BCI:1.52- 2.47, *p* < 0.001). In the 80 + group the confidence interval indicate compatibility with a small-moderate harmful effect of weak HGS in the probable OSA group than protective effect (OR:1.22, BCI: 0.86- 1.74, *p* = 0.252). In males, probable OSA was associated with an 87% increase in odds of weak HGS (OR = 1.87, BCI:1.49–2.35, *p* < 0.001) while in females, the odds were 2.7 times higher (OR = 2.67, BCI 2.15–3.33, *p* < 0.001).

## Discussion

This study investigated the cross-sectional associations between probable (high-risk) OSA, derived using an adapted STOP-Bang score, and poor physical performance in standing balance, gait speed and relative HGS, in a large, representative sample of adults over 50 years of age. Consistent with our hypothesis, we found that probable OSA is associated with: (i) poor standing balance, (ii) slow gait speed, and (iii) weak HGS in the overall sample.

Among middle-aged adults (50–64 years), those with probable OSA had a 41% greater odds of poor balance and more than threefold increase in odds of weak HGS. While impaired balance and weak muscle strength typically progress as we age [40], these findings suggest that neuromuscular impairments associated with OSA may manifest earlier, during midlife, before overt frailty is clinically apparent. Midlife may therefore represent a critical window for early identification and intervention to prevent downstream physical decline and falls in people at high risk of OSA [41].

In adults aged 65–79 years, associations between OSA risk and balance or gait were attenuated and no longer statistically significant, though confidence intervals remained compatible with small to moderate adverse effects. In contrast, those with probable OSA had 93% increased odds of having weak HGS. These findings may reflect the increasing influence of age-related sarcopenia, multimorbidity, and other non-OSA-related contributors to functional decline that dilute the observable effect of OSA on balance and gait [42]. Muscle strength, however, may remain a more sensitive marker of OSA-related neuromuscular burden in this age group, aligning with evidence that intermittent hypoxia impairs muscle protein synthesis and accelerates muscle weakness independently of other ageing processes [43].

Among adults aged 80 years and older, the association of probable OSA with poor balance and weak HGS was uncertain, whereas the strongest observed association was with slow gait speed, showing a 61% higher odds. This may reflect a cumulative burden of OSA-related pathophysiological stressors over time, including chronic intermittent hypoxia, cerebrovascular dysregulation, and impaired sensory integration that specifically compromise gait [8]. The lack of significant associations with balance and HGS, along with wide BCI in the association with gait speed may partly reflect healthy survivor bias, residual confounding from multimorbidity and frailty, and competing age-related functional decline, which could attenuate or obscure the observable impact of OSA on these outcomes.

When stratified by sex, probable OSA was significantly associated with poor balance, slow gait speed and weak HGS in females, with weaker and non-significant associations observed in males. Although sex-specific biological mechanisms were not explicitly examined in this study, the stronger associations in females may partly reflect sex-specific OSA pathophysiology, including greater sleep fragmentation, prolonged partial airway obstruction, and more frequent symptoms such as fatigue and mood disturbances despite lower apnea hypopnea index (AHI) than males [44, 45]. Hormonal and inflammatory factors, such as oestrogen decline and systemic inflammation, may further increase neuromuscular vulnerability in females [44], potentially explaining the stronger associations observed in our study.

The associations observed between OSA and poorer physical performance in the present study align with emerging evidence that sleep-disordered breathing may impair balance, gait, and muscle strength in older adults accelerating age-related functional decline and thereby increasing fall risk [46]. While causality cannot be inferred, several pathophysiological mechanisms underlying OSA have been proposed to increase the risk of physical performance deficits. These include chronic intermittent hypoxia and sleep fragmentation, which may impair sensory integration, including visual, vestibular, and proprioceptive inputs required for balance [47], disrupt cerebral autoregulation and brain regions involved in gait coordination [48], and reduce anabolic hormone production, essential to maintain muscle strength [8, 49]. However, given the cross-sectional nature of the study, reverse causality is also possible whereby age-related sarcopenia and reduced muscle strength may increase the risk of OSA [20]. Future studies are needed to clarify these mechanisms and inform targeted interventions.

Our findings reinforce and extend prior research linking OSA with impaired postural balance and gait, and reduced muscle strength. Previous studies with small, clinic-based samples have demonstrated that OSA is associated with postural instability, including increased mediolateral sway and faster centre-of-gravity displacement [13–15], reduced gait speed, increased stride variability, and wider steps [19, 50] and that individuals with severe OSA (AHI > 30) have reduced handgrip strength [20]. While these findings are important, our study builds on this literature in several novel ways. A key strength of this study is the use of a representative population-based sample of older adults, and the assessment of multiple domains of physical performance, i.e., standing balance, gait speed, and relative handgrip strength, which enhances the generalisability of our findings to community-living populations and a comprehensive, objective evaluation of fall-related functional outcomes. Importantly, we employed the validated and widely used STOP-Bang questionnaire, with high specificity [27], allowing us to capture data in those at high risk of OSA. In this study, handgrip strength was normalized to BMI, which accounts for differences in body size, which is particularly important in older adults who experience changes in body composition with ageing [9, 10].

However, several limitations warrant consideration. The cross-sectional design limits causal inference. We lacked objective sleep measurements, and gait data were unavailable for participants under 65 years of age. Residual confounding, especially from unmeasured comorbidities and hormone replacement cannot be ruled out. Although the observed associations were statistically significant, the magnitude of the effects was modest, and these findings should not be interpreted as large clinically actionable differences at the individual level, rather as an indication of association. Additionally, given the reduced sensitivity of STOP-Bang questionnaire screening in women [51], some female participants with OSA may have been misclassified as low risk, which would bias associations toward the null. Yet, the significant associations observed regardless of the bias suggest robustness of the finding.

## Conclusion

A high risk of OSA is associated with poorer physical performance in balance, gait and muscle strength, with evidence of this association observed even in midlife adults aged 50–64 years. Given that most fall prevention efforts have focused on adults over 60 years of age, these findings highlight the need for further investigation into when OSA-related changes in physical performance may begin, to inform earlier and more effective fall prevention strategies. This study provides preliminary evidence linking OSA to impaired physical performance and future studies are required to evaluate causal relationships to determine if co-screening approaches for OSA and fall-related impairments in midlife adults may help guide timely, targeted interventions to preserve physical function and reduce future fall risk.

## Supplementary Information

Below is the link to the electronic supplementary material.

Supplementary file1 (DOCX 23 KB)

## Data Availability

Data for the Health and retirement study public survey are available at https://hrsdata.isr.umich.edu/data-products/public-survey-data?_gl=1*1axbmvs*_ga*MTc4NDQ5NTcwMS4xNzM3MzQwNzkx*_ga_FF28MW3MW2*czE3NjIzMDAxNDYkbzQxJGcxJHQxNzYyMzAwMTUzJGo1MyRsMCRoMA.
