# Supplementary material for: Obstructive sleep apnea risk is associated with poor physical performance: a cross-sectional analysis of the U.S. health and retirement study
Source: J Clin Sleep Med. 2026 Apr 7;22(1):46. doi: 10.1007/s44470-026-00070-2 (PMC13057046; doi:10.1007/s44470-026-00070-2)
Supplement: Supplementary file 1 — Supplementary file1 (DOCX 23 KB) [file 44470_2026_70_MOESM1_ESM.docx]

**Obstructive sleep apnea risk is associated with poor physical performance: A cross-sectional analysis of the U.S. Health and Retirement study**

# **S1. Sensitivity analysis:**

Binomial logistic regression analyses examined associations of OSA risk as an independent variable with standing balance, gait speed and relative HGS as dependent variables in participants who were untreated for any sleep/ snoring problems. Models for balance and gait speed were adjusted for age, sex, BMI, race, alcohol consumption, comorbidity burden, treatment for sleep/ snoring problem and LTPA. The model for relative HGS was not adjusted for BMI since the outcome already included BMI. All models were further examined using age and sex-stratified analyses. Bootstrapped confidence intervals (BCI;95%) were used to improve the precision and reliability of the estimated association.

### **OSA and balance stratified by age and sex**

Table I shows the odds of poor balance in the high-risk OSA group across different age groups and sexes. The results are consistent with main analysis.

Table I: Odds of poor balance in high-risk OSA

| **Stratification** | **Category** | **Sample size** | **OR** | **Bootstrapped 95% CI** | **p-value** |
| --- | --- | --- | --- | --- | --- |
| Overall | All | Total: 7039;  Low Risk: 4837;  High Risk: 2202 | 1.20 | 1.05 – 1.37 | **0.008** |
| Age Group | 50-64 | Total: 3567;  Low Risk: 2066;  High Risk: 1501 | 1.41 | 1.09 – 1.66 | **0.001** |
| Age Group | 65-79 | Total: 2232;  Low Risk: 1372; High Risk: 860 | 1.13 | 0.92 – 1.42 | 0.257 |
| Age Group | 80+ | Total: 803;  Low Risk: 582; High Risk: 221 | 0.85 | 0.58 – 1.27 | 0.402 |
| Sex | Male | Total: 2852;  Low Risk: 1294; High Risk: 1558 | 1.16 | 0.91 – 1.42 | 0.127 |
| Sex | Female | Total: 3750;  Low Risk: 2726; High Risk: 1024 | 1.31 | 1.07 – 1.59 | **0.003** |

### **OSA and gait stratified by age and sex**

Table II shows the odds of slow gait speed in the high-risk OSA group across different age groups and sexes. For age group 80+ year, the CI is more compatible with harmful effect of high-risk OSA on gait speed rather than any protective effect, which is consistent with our main analysis despite not meeting the traditional significance level of p<0.05. The wide CI indicates we may not have enough power to detect that association after reducing total number of participants in this subgroup. Other results are consistent with our main findings.

Table II:Odds of slow gait speed in high-risk OSA

| **Stratification** | **Category** | **Sample size** | **OR** | **Bootstrapped 95% CI** | **p-value** |
| --- | --- | --- | --- | --- | --- |
| Overall | All | Total: 2850;  Low Risk: 1858; High Risk: 992 | 1.23 | 1.00 – 1.51 | **0.040** |
| Age Group | 65-79 | Total: 2030;  Low Risk: 1281; High Risk: 749 | 1.10 | 0.87 – 1.37 | 0.391 |
| Age Group | 80+ | Total: 797;  Low Risk: 576; High Risk: 221 | 1.44 | 0.97 – 2.25 | 0.098 |
| Sex | Male | Total: 1166;  Low Risk: 577; High Risk: 589 | 1.12 | 0.86 – 1.48 | 0.398 |
| Sex | Female | Total: 1661;  Low Risk: 1280; High Risk: 381 | 1.38 | 1.02 – 1.92 | **0.033** |

### OSA and relative HGS stratified by age and sex

Table III shows the odds of poor balance in the high-risk OSA group across different age groups and sexes. The results are consistent with main analysis.

Table III: Odds of weak HGS in high-risk OSA

| **Stratification** | **Category** | **Sample Size** | **OR** | **95% Bootstrap CI** | **p-value** |
| --- | --- | --- | --- | --- | --- |
| Overall | All | 6814 | 2.25 | 1.79 – 2.52 | **<0.001** |
| Age Group | 50-64 | Total: 3215;  Low Risk: 1938;  High Risk: 1277 | 3.35 | 2.51 – 4.56 | **<0.001** |
| Age Group | 65-79 | Total: 2077;  Low Risk: 1291; High Risk: 786 | 1.84 | 1.42 – 2.34 | **<0.001** |
| Age Group | 80+ | Total: 811;  Low Risk: 584; High Risk: 227 | 1.15 | 0.80 – 1.61 | 0.441 |
| Sex | Male | Total: 2608;  Low Risk: 1232; High Risk: 1376 | 1.78 | 1.40 – 2.25 | **<0.001** |
| Sex | Female | Total: 3495;  Low Risk: 2581; High Risk: 914 | 2.54 | 2.02 – 3.18 | **<0.001** |
